# Grazing Responses of Distinct-Sized Tropical Cladocerans to Different Filamentous Sizes of the Cyanobacterium *Dolichospermum planctonicum*

**DOI:** 10.3390/microorganisms14030590

**Published:** 2026-03-06

**Authors:** Luciana Machado Rangel, Larissa Ramos Ribeiro, João Paulo Santana Valério, Marcelo Manzi Marinho, Marcella Coelho Berjante Mesquita

**Affiliations:** Laboratory of Ecology and Physiology of Phytoplankton, Department of Plant Biology, University of Rio de Janeiro State, Rua São Francisco Xavier 524-PHLC Sala 511, Rio de Janeiro 20550-900, Brazil; ramoslarissa086@gmail.com (L.R.R.); valeriojoao1@hotmail.com (J.P.S.V.); manzi.uerj@gmail.com (M.M.M.)

**Keywords:** harmful algal blooms, trophic interactions, tropical zooplankton, eutrophication, feeding inhibition

## Abstract

Cyanobacterial blooms directly influence the structure and function of zooplankton communities; however, the trophic interactions between small tropical cladocerans and the cyanobacterium *Dolichospermum* are still poorly understood. We evaluated how two strains of *Dolichospermum planctonicum* (differing in filament length) affect the grazing rates of three tropical cladocerans with distinct size and prey spectra—*Daphnia gessneri*, *Ceriodaphnia silvestrii*, and *Macrothrix paulensis*—in single and mixed diets with the chlorophyte *Monoraphidium capricornutum*. Overall, grazing rates decreased as food concentration increased across all phytoplankton species. *Daphnia* was the most efficient filter-feeder in all diets, yet the responses to different-sized *Dolichospermum* strains varied between animals and diets. Shorter *Dolichospermum* was the least consumed food item in single diets, as opposed to what was observed in the mixed diets, where it was the most consumed. This reversal suggests that the mechanism limiting grazing on *Dolichospermum* might change drastically depending on the food context (availability of other food sources). Positive selectivity for both *Dolichospermum* and *Monoraphidium* was observed for all cladocerans. These findings highlight that the morphology of *Dolichospermum planctonicum* and the availability of alternative food sources during its blooms are critical regulators of grazing. The results also provide evidence of diverse feeding strategies of tropical cladocerans to prey on the filamentous cyanobacterium *Dolichospermum planctonicum.*

## 1. Introduction

Intensified anthropogenic activities have degraded aquatic ecosystems into a global environmental crisis, directly threatening public health, depleting biodiversity, and compromising economic activities by reducing the supply of safe drinking water [1,2]. Anthropogenic eutrophication, driven by high nitrogen (N) and phosphorus (P) loads from agriculture, untreated sewage, and industrial effluents, creates optimal conditions for cyanobacterial blooms in freshwater systems [3,4]. Currently, global climate change is also modulating the occurrence patterns of these events [5,6]. Rising average water temperatures and extended thermal stratification provide selective advantages to cyanobacteria over other phytoplankton taxa [7,8]. Furthermore, ecophysiological traits, such as very efficient nutrient uptake and storage strategies, facilitate cyanobacterial dominance across diverse environmental gradients [9,10,11,12]. Evidence suggests that bloom-forming species also experience significantly lower top-down pressure compared to other taxa [13,14]. This reduced grazing pressure, often attributed to the production of harmful secondary metabolites, complex morphology, and high biomass, is a key factor in the ecological success and persistence of these species [15,16,17].

The rising frequency and intensity of these blooms have intensified research into cyanobacteria–zooplankton interactions [13,18]. Harmful cyanobacteria adversely affect zooplankton through low palatability, morphological constraints, and toxicity, impacting feeding rates, reproductive success, and survival [12,19,20]. Nevertheless, certain zooplankton populations, especially those with a history of coexistence with toxic blooms, may exhibit enhanced tolerance [14,21]. While current research predominantly focuses on large-bodied cladocerans such as *Daphnia*, there remains a critical scarcity of information regarding interactions between smaller cladocerans and bloom-forming cyanobacteria [13,14,22].

In tropical ecosystems, toxic blooms exert direct selective pressure on zooplankton community structure, often inducing a shift in body size distribution where large-bodied species are replaced by smaller organisms, such as rotifers and small cladocerans (e.g., *Ceriodaphnia*, *Macrothrix*, and *Moina*) [23,24]. These smaller taxa may exhibit distinct ecological responses and escape mechanisms to the deleterious effects of blooms [14,25,26]. While the pelagic cladocerans *Daphnia* and *Ceriodaphnia* are characterized as generalist filter-feeders that rely on passive filtration, the typically littoral *Macrothrix* exhibits more specialized feeding strategies, including scraping and the selective capture of particles [27].

Among the most prevalent bloom-forming genera, *Dolichospermum* stands out due to its metabolic versatility, producing potent cyanotoxins, including microcystins, cylindrospermopsins, anatoxin-a, guanitoxin, and saxitoxins [4,28]. The genus can also produce volatile organic compounds (VOCs) like 2-methylisoborneol (MIB) and geosmin [29,30]. *Dolichospermum* species are characterized by unbranched filaments (trichomes) that exhibit significant morphological variation, ranging from straight to coiled and irregularly twisted, which can appear as solitary individuals or complex aggregates [28]. Additionally, the impact of zooplankton grazing on the species of *Dolichospermum* in the tropics remains poorly characterized, representing a substantial knowledge gap regarding top-down control mechanisms.

Thus, this study aimed to evaluate the variability in grazing rates of three tropical cladoceran species (*Daphnia gessneri*, *Ceriodaphnia silvestrii* and *Macrothrix paulensis*) with distinct body sizes and prey spectra in response to two strains of *Dolichospermum planctonicum* in single and mixed diets in combination with the high-quality chlorophyte *Monoraphidium capricornutum*. The zooplankton clones have different histories of exposure to cyanobacteria blooms as they were isolated from two Brazilian reservoirs with a documented record of cyanobacterial blooms: the Funil reservoir [7] and the Juturnaíba reservoir [2,31]. The *D. planctonicum* strains used in the study were isolated from the Juturnaíba reservoir.

Specifically, this study aims to evaluate: (i) how varying concentrations of *D. planctonicum* strains with different morphologies impact the filtration rates of tropical cladocerans with distinct body sizes and prey-size spectra; (ii) to what extent the proportions of different *D. planctonicum* strains within a mixed diet, with a high-quality food source also present, influence the grazing dynamics of tropical cladocerans occupying diverse feeding niches; and (iii) the selective feeding patterns of tropical cladocerans with a history of cyanobacteria exposure when offered a choice between filamentous *D. planctonicum* and the nutritionally superior green alga *M. capricornutum*.

We hypothesized that: (i) grazing will decrease significantly as the concentration and filament size of *D. planctonicum* increase, with larger cladoceran species exhibiting a more pronounced reduction in filtration due to mechanical interference (clogging) of their filtering apparatus compared to smaller species; (ii) the grazing dynamics will be non-linearly affected by the proportion of cyanobacteria, and, specifically, a ‘threshold’ effect is expected where high proportions of long-filament strains will suppress the ingestion of *M. capricornutum* across all feeding niches, but with greater resilience in species with wider prey-size spectra; and (iii) cladocerans with a history of exposure to *D. planctonicum* will exhibit a strong selective preference for *M. capricornutum* and avoid *D. planctonicum* filaments, but this selectivity will be less efficient when filament morphology closely mimics the size of the high-quality food source.

## 2. Materials and Methods

### 2.1. Phytoplankton Cultivation

Two distinct strains of the filamentous cyanobacterium *Dolichospermum planctonicum* (DPJ-01 and DPJ-07) were used in this study. Strains were isolated from the Juturnaíba reservoir (Rio de Janeiro, Brazil) between 2024 and 2025 from single filaments of natural population samples using the micropipetting and serial dilution technique. Both strains exhibited straight filaments, though with significant differences in mean filament size, which may influence grazing loss rates [32]. During the experimental period, DPJ-01 strain cultures showed a length and width of 224.67 (±196.7) μm × 8.97 (±1.2) μm, while filaments from DPJ-07 strain cultures were larger, with a length and width of 636.55 (±665.1) μm × 9.97 (±0.8) μm. The Mann–Whitney test indicated significant differences in the average filament sizes of the strains (U = 673, *p* ≤ 0.05), but not in cell size.

The chlorophyte *Monoraphidium capricornutum* (diameter 2–4 μm; length 9–18 μm) was used as a high-quality food source for zooplankton. This unicellular species was selected for its appropriate cell size and was kindly provided by the Laboratory of Ecophysiology and Toxicology of Cyanobacteria (Federal University of Rio de Janeiro). Previous studies have demonstrated this chlorophyte to be of high nutritional quality for the growth and survival of cladocerans [19,20].

Experimental phytoplankton cultures were maintained in climate-controlled chambers at a constant temperature of 24 °C, with a light intensity of ≈40 µmol photons m^−2^ s^−1^ and a 12:12 h light/dark photoperiod. Modified WC medium [33] was used for cultivation. To ensure cultures remained in the exponential growth phase, the medium was renewed weekly with fresh, sterilized medium.

### 2.2. Zooplankton Cultivation

Three cladoceran species were isolated from a single female of each species and maintained in clonal cultures to ensure genetic uniformity: *Daphnia gessneri* (isolated from Funil reservoir—Rio de Janeiro, Brazil), *Ceriodaphnia silvestrii*, and *Macrothrix paulensis* (isolated from Juturnaíba reservoir—Rio de Janeiro, Brazil). The Funil reservoir [7] and the Juturnaíba reservoir [2,31] have recorded diverse events of cyanobacterial blooms. In the year in which cladocerans and cyanobacteria were isolated from these waterbodies, the Funil reservoir was dominated by *Microcystis aeruginosa* and presented reduced biomass of *Dolichospermum cicinale*, and the Juturnaíba reservoir was dominated by *Dolichospermum planctonicum* throughout the study year (data in preparation). These species are common in Neotropical waterbodies and are predominantly filter-feeders [34], though they occupy different niches: *D. gessneri* and *C. silvestrii* are generalist pelagic filter-feeders, while *M. paulensis* exhibits detritivorous tendencies and is associated with benthic substrates and macrophyte surfaces [27].

Organisms were cultured in 1–2 L beakers containing a 90:10 mixture of artificial RT medium [35] and filtered, autoclaved water from the Juturnaíba reservoir. Cladocerans were maintained at 24 °C with a 12:12 h light/dark cycle in incubators SOLAB SL-224 (SOLAB, Piracicaba, Brazil). They were fed *M. capricornutum* three times a week at a total carbon concentration of 0.5 mg C L^−1^. Cultivation media were completely renewed weekly.

During the experimental period, the length and width of *D. gessneri*, *C. silvestrii*, and *M. paulensis* were, respectively, 1280 (±93) μm × 682 (±70) μm; 660 (±76) μm × 520 (±63) μm; and 636 (±66) μm × 356 (±56) μm (n = 10). One-way ANOVA identified that the length and width dimensions of the three animals were significantly different (length: F_2,29_ = 256.44, *p* < 0.001; width: F_2,29_ = 66.42, *p* < 0.001).

### 2.3. Grazing Experiments

#### 2.3.1. Single Diets at Varying Concentrations

The single-diet grazing experiment aimed to evaluate the feeding behavior of three cladoceran clones with different sizes and food spectra in response to different concentrations of algal suspension from two *Dolichospermum planctonicum* strains (DPJ-01 and DPJ-07), as well as a nutritive food source (the chlorophyte *Monoraphidium capricornutum*), to investigate whether these different diets at different concentrations influence the feeding behavior of tropical cladocerans. Five carbon-based concentrations (0.125, 0.25, 0.5, 1.0 and 2.0 mg C L^−1^) were used for each algal suspension.

To estimate the total carbon content of the experiments, cell counts of the algal cultures were performed to estimate cell density, followed by conversion to carbon and its respective dilutions. Carbon estimation in algal cultures was based on biovolume, applying the conversion formula of C = aV^b^ (where C is carbon, V is algal volume, and the constants are a = 0.1204 and b = 1.051) [36]. To achieve the required carbon dilutions, the cultures were diluted with modified WC medium [33].

Adult cladocerans were transferred to a beaker containing only their culture medium, 12 h before the start of the experiment, and kept fasting during this period (without the addition of food). The 12 h interval was established to ensure the complete elimination of the contents present in the gastrointestinal tract of the animals, ensuring that there was no prior food interference in the subsequent analyses. To determine the intestinal emptying time, preliminary tests were carried out. In these trials, the cladocerans were fed at the beginning of the day and then observed every hour until the complete evacuation of the digestive contents.

The experimental grazing assays were carried out in 24-well culture plates, filling each well with 2.5 mL of the food suspension. The plates were subsequently incubated in the dark under controlled temperature conditions (24 ± 1 °C) for a period of 3 h. Darkness conditions are implemented to minimize chlorophyll variations not derived from grazing [37]. Both treatments and controls were replicated four times. Each treatment replicate contained three adult individuals of a given cladoceran species per well, with experiments for the different species conducted separately, without coexistence of distinct species in the same assay. The control wells contained only food suspension, without the presence of the animals.

The clearance rates of the cladocerans (CR, in mL ind^−1^ h^−1^) were estimated by the difference in chlorophyll-a concentrations using a Phyto-PAM (Phytoplankton Analyzer, Heins-WALZ, Effeltrich, Germany), according to the methodology proposed by [37]. Differential quantification of phytoplankton groups (cyanobacteria and chlorophyta) can be achieved through this equipment. This system utilizes four distinct excitation wavelengths (470, 535, 620, and 659 nm) to deconvolve taxonomically specific fluorescence signals based on varying accessory pigment composition. The measuring LED array comprises 25 LEDs for fluorescence induction and 12 actinic LEDs (peaking at 655 nm) [38]. As described in [37], only the measuring light was employed, delivered in microsecond pulses at a low intensity of 1 μmol photons m^−2^ s^−1^. This minimal irradiance prevents electron accumulation in Photosystem II. Consequently, the measured values represent the minimum fluorescence yield (F_0_) of dark-adapted samples. As F_0_ is independent of active photosynthetic electron transport, it serves as a robust proxy for chlorophyll-a concentration [37,38].

To account for strain-specific optical properties, reference spectra were generated for each phytoplankton strain according to the manufacturer’s protocols and applied to all experiments. Samples were dark-adapted prior to analysis and transferred to the cuvette under dim light conditions. As the fluorescence signal reached a steady state, and considering the brief duration required for data acquisition, cellular sedimentation was deemed negligible. Furthermore, considering the higher rates of sedimentation and aggregation of filamentous algae, an integrated miniature magnetic stirrer was utilized during the measurements [38]. For chlorophyll calibration, a sample of pure culture of each phytoplankton strain used in the experiment was measured through the Biospectro spectrophotometer, model SP-22 (Biospectro, Curitiba, Brazil), after extraction in 90% acetone, according to the method described in [39]. The values obtained were then measured and added to Phyto-PAM, according to the manufacturer’s instructions.

After the reference and calibration steps, chlorophyll concentrations from grazing experiments were read in Phyto-PAM before and after the incubation period. The equation described below, proposed by [40], was used to calculate the CRs:

CR = {ln(Chla_control_ − Chla_treataments_)} × (V/N),

where

Chla_control_: average final algal concentration in the control;

Chla_treatment_: average final algal concentration in the treatment;

V: culture volume (mL);

N: number of cladocerans per well.

The coefficients Chla_control_ and Chla_treatments_ were calculated as:

Chla_control_: ((ln(A_C,t1_) − (ln(A_C,t0_))/∆t

and

Chla_treatments_: ((ln(A_T,t1_) − (ln(A_T,t0_))/∆t

In these equations, A_C,t0_ and A_C,t1_ represent the initial and final algal concentrations in the experimental controls. Similarly, A_T,t0_ and A_T,t1_ denote the initial and final algal concentrations in the experimental treatments. Δt indicates the total incubation duration (h).

In addition, as described in [37], non-target constituents besides phytoplankton (such as WC compounds) may contribute to the total fluorescence signal. To isolate the signal originating exclusively from phytoplankton chlorophyll, a digital Zero-offset (Zoff) correction was applied. Also, soluble fluorescing compounds are expected to occur in the treatments due to active grazing on phytoplankton, as the pigments are released during cell lysis and digestion. Therefore, a correction to this was performed by analyzing the filtrate of each sample passed through a 0.45 m membrane filter, as suggested by [37] and also successfully carried out by [14,15,21,41,42,43]. In a few cases, the results of the chlorophyll difference between controls and treatments were equal to or less than 0. In such cases, the experiments were repeated.

To ensure a homogenous distribution of the prey community and prevent the sedimentation of pytoplankton, all food suspensions were subjected to gentle aeration via brief bubbling with a Pasteur pipette once every hour. The physiological status and motility of cladocerans were also monitored hourly throughout the incubation period to ensure consistent grazing activity. Upon completion of the grazing interval, all grazers were promptly removed from the experimental units to avoid interference during the subsequent fluorometric analysis [21,37].

#### 2.3.2. Mixed Diets

With the aim of evaluating the effects of the relative dominance of cyanobacteria in the feeding of zooplanktonic organisms, grazing experiments were carried out using different clones of cladocerans fed with mixed diets, which were composed of increasing proportions of *Dolichospermum planctonicum* strains (DPJ-01 or DPJ-07) in different combinations with the chlorophycean *Monoraphidium capricornutum*. Cyanobacteria were added to the diets in three distinct proportions—25%, 50% and 75%—and were complemented by *Monoraphidium capricornutum* in the respective proportions of 75%, 50% and 25%, maintaining a constant total food concentration of 0.5 mg C L^−1^.

This experimental design aimed to understand how the dominance of cyanobacteria in mixed diets influences the filtration rate of: (A) *Dolichospermum planctonicum* (CRBlue), (B) *Monoraphidium capricornutum* (CRGreen), and (C) the combined total of the foods (CRTotal). Each treatment was performed with four replicates, following the same procedures adopted in the experiments with single diets, described previously.

#### 2.3.3. Selectivity Index (α)

From the results of the mixed diets, the selectivity coefficient (α) (α = Igreen algae/(Igreen algae + Icyanobacteria)) was calculated for each type of food, using the normalized Ivlev ratio, based on variations in chlorophyll-a concentration, according to the methodology described by [44]. The value of coefficient α varies between 0 and 1, with values greater than 0.5 indicating positive selection by the food, values equal to 0.5 suggesting a lack of selectivity, and values less than 0.5 indicating rejection of the food by the organisms.

### 2.4. Statistical Analysis

Filament size differences were analyzed using the Mann–Whitney Rank Sum Test. Body size differences were assessed via one-way ANOVA with Holm–Sidak post hoc comparisons (SigmaPlot v.14). The effects of cladoceran species, diet type, and concentration/proportion on clearance rates were evaluated using Generalized Linear Models (GLMs) with a Gaussian family function in R (v.4.5.1), utilizing the *vegan* and *emmeans* packages. A three-way ANOVA was performed for selectivity indices.

## 3. Results

### 3.1. Single-Diet Experiments at Varying Concentrations

**Figure 1:**
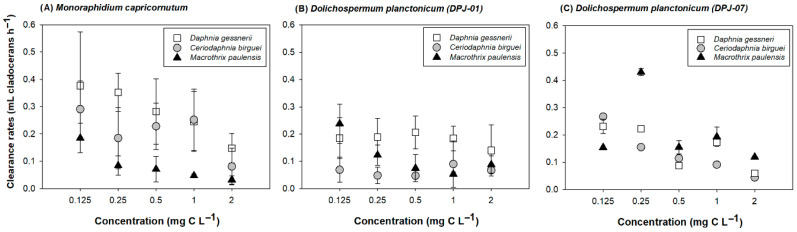
Clearance rates of the studied cladocerans in single-diet experiments across different concentrations of *Monoraphidium capricornutum* and *Dolichospermum planctonicum* strains (DPJ-01 and DPJ-07). Error bars indicate standard deviation (n = 4).

Clearance rates in single-diet experiments exhibited considerable variation (Figure 1). The highest grazing rate was recorded for *Daphnia gessneri* fed with *Monoraphidium capricornutum* at a concentration of 0.125 mg C L^−1^ (0.37 ± 0.16 mL ind^−1^ h^−1^), while the lowest was observed for *Macrothrix paulensis* under the same diet but at a 2 mg C L^−1^ concentration (0.03 ± 0.02 mL ind^−1^ h^−1^).

Experimental results (GLM analyses) showed that zooplankton species (F_2,179_ = 12.7; *p* < 0.001), cyanobacteria strains (F_2,179_ = 8.8; *p* < 0.001), and food concentration (F_4,179_ = 11.8; *p* < 0.001) all played a decisive role in clearance rates. *Daphnia gessneri* emerged as the most efficient grazer, significantly outperforming *Ceriodaphnia silvestrii* and *Macrothrix paulensis* (Table 1). When exposed to different diets, the zooplankton consumed notably less *Dolichospermum planctonicum* DPJ-01 compared to *Monoraphidium capricornutum* or the DPJ-07 strain. Additionally, a consistent pattern emerged across all groups: as food became more abundant, individual consumption rates steadily declined (Figure 1; Table 1).

### 3.2. Mixed-Diet Experiments with Varying Proportions of Cyanobacteria

**Figure 2:**
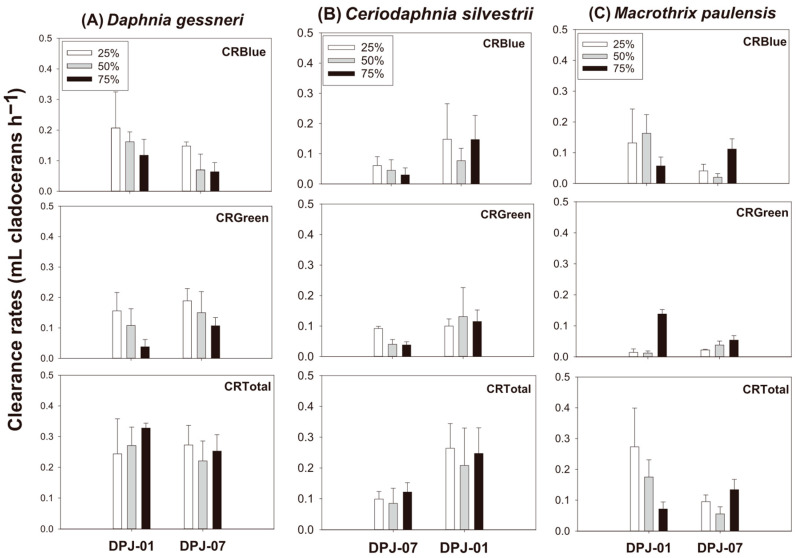
Clearance rates of cladocerans on *Dolichospermum planctonicum* (DPJ-01 and DPJ-07), *Monoraphidium capricornutum*, and total food (combined) at varying dietary proportions of cyanobacteria (25%, 50%, and 75%). Error bars indicate standard deviation (n = 4).

Clearance rates on *Dolichospermum planctonicum* were primarily driven by the zooplankton species (F_2,71_ = 7.6; *p* < 0.05) and the specific strains provided (F_2,71_ = 24.2; *p* < 0.001). *Daphnia gessneri* maintained notably higher clearance rates than *Ceriodaphnia silvestrii*, demonstrating a greater grazing capacity. In contrast, the proportion of cyanobacteria in the diet did not significantly influence consumption rates. Among the cyanobacterial diets (CRBlue), the specific strain proved to be the most critical factor in determining feeding intensity. Regarding strains, clearance rates on DPJ-01 diets were significantly higher than those on DPJ-07 (Figure 2; Table 2). As for *Monoraphidium capricornutum* clearance rates within mixed diets (CRGreen), significant differences were observed only for the zooplankton species (F_2,71_ = 11.3; *p* < 0.001), with *Macrothrix paulensis* exhibiting significantly lower rates compared to the other cladocerans. Total clearance rates (combined cyanobacteria and green algae—CRTotal) differed significantly by zooplankton species (F_2,71_ = 15.7; *p* < 0.001) and strains (F_2,71_ = 25.9; *p* < 0.001). *Daphnia gessneri* maintained higher total rates than the other species, and total consumption was significantly more impacted by DPJ-01 than DPJ-07 (Figure 2; Table 2).

### 3.3. Selectivity Indices

**Figure 3:**
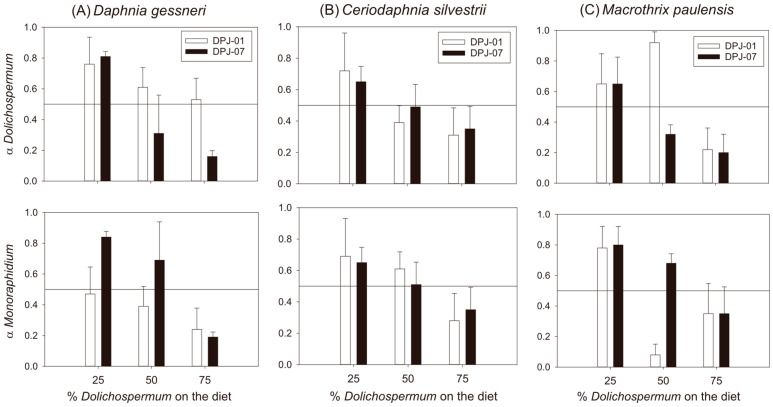
Mean selectivity coefficients (α) of cladocerans for *Dolichospermum planctonicum* and *Monoraphidium capricornutum* in mixed diets. The solid line at α = 0.5 represents the threshold for positive selection. Error bars indicate standard deviation (n = 4).

Positive selectivity (selectivity coefficient α > 0.5) for both *Monoraphidium capricornutum* and *Dolichospermum planctonicum* strains occurred primarily in diets with lower cyanobacterial proportions (25% and 50%). Conversely, negative selectivity for both food types was observed mainly at the 75% cyanobacterial proportion (Figure 3). Selectivity for both *Dolichospermum planctonicum* and *Monoraphidium capricornutum* was significantly shaped by the specific strain and the proportion of cyanobacteria in the diet. For *Dolichospermum planctonicum*, both strain (F_1,71_ = 13.4, *p* < 0.001) and proportion (F_1,71_ = 46.4, *p* < 0.001) were key drivers. Specifically, grazers showed a higher selectivity for *Dolichospermum planctonicum* DPJ-01 (t = 3.6, *p* < 0.001), though this preference weakened as the cyanobacterial proportion increased (25% vs. 75%: t = 9.6, *p* < 0.001; 50% vs. 75%: t = 5.1, *p* < 0.001). Similarly, for *Monoraphidium capricornutum*, selectivity was influenced by strain (F_1,71_ = 14.1, *p* < 0.001) and proportion (F_1,71_ = 42.1, *p* < 0.001), with *Dolichospermum planctonicum* DPJ-07 treatments evoking higher selectivity (t = 3.8, *p* < 0.001). Again, an increase in cyanobacterial proportion negatively affected selectivity (Figure 3). Furthermore, a significant three-way interaction between zooplankton species, strain, and proportion was observed for both *Dolichospermum planctonicum* (F_4,71_ = 5.1, *p* < 0.001) and *Monoraphidium capricornutum* (F_4,71_ = 5.5, *p* < 0.001), suggesting that grazing preferences are complex and context-dependent. Holm–Sidak post hoc analysis revealed higher selectivity for *Dolichospermum planctonicum* DPJ-01 (t = 3.6, *p* < 0.001) and a reduction in selectivity as the cyanobacterial proportion increased (25% vs. 75%: t = 9.6, *p* < 0.001; 50% vs. 75%: t = 5.1, *p* < 0.001). For *Monoraphidium capricornutum*, selectivity was higher in treatments involving *Dolichospermum planctonicum* DPJ-07 (t = 3.8, *p* < 0.001) and was similarly negatively affected by increasing cyanobacterial proportions in the diet (Figure 3).

## 4. Discussion

In this study, we evaluated the variations in grazing responses of three tropical cladocerans with distinct body sizes and prey spectra in response to two strains of *Dolichospermum planctonicum*. In single diets, we can observe mechanisms related to a single available food source and how the concentration of each source (whether chlorophyte *Monoraphidium capricornutum* or small or large *D. planctonicum*) affects cladocerans with distinct grazing strategies. In mixed diets combining *D. planctonicum* in different proportions with chlorophyte *M. capricornutum*, we aimed to understand mechanisms that act under different dominance conditions, mimicking the onset, growth and established bloom. Our findings demonstrate that the grazing of tropical cladoceran on *D. planctonicum* is strain-specific and dependent on the consumer cladoceran species. Furthermore, food concentration and dietary proportion of cyanobacteria significantly modulate feeding behavior.

*How do varying concentrations of* *D. planctonicum* *strains with different morphologies impact the filtration rates of tropical cladocerans with distinct body sizes and prey-size spectra?*

A primary finding was the variability in grazing efficiency among cladocerans of different sizes. The largest species, *Daphnia gessneri*, exhibited the highest efficiency in consuming the green alga *Monoraphidium capricornutum*, yet its clearance rate was severely inhibited by the long-filament strain of *D. planctonicum* (DPJ-07) in single diets. In contrast, the smallest species, *Macrothrix paulensis*, showed higher tolerance and the highest clearance rates in the presence of this cyanobacterium. *Ceriodaphnia silvestrii* displayed intermediate clearance rates and a tendency for greater inhibition by the DPJ-01 strain. All three species showed reduced clearance rates as food concentration increased in the single-diet assays, aligning with conceptual models where eutrophication and high phytoplankton biomass reduce energy transfer efficiency within food webs [1,13,45].

These results suggest that tropical cladocerans, often classified as generalist filter-feeders, can vary significantly in habitat and feeding strategy. While *Daphnia gessneri* and *Ceriodaphnia silvestrii* are pelagic and believed to rely on passive filtration feeding behavior, *Macrothrix paulensis* is typically littoral and capable of scraping or selectively capturing particles [27]. Our results align with the hypothesis that zooplankton–cyanobacteria interactions are complex and mediated by mechanical, nutritional, and chemical factors [14,25,46]. The marked reduction in *Daphnia gessneri* grazing on the longest *Dolichospermum planctonicum* strain (DPJ-07; mean length = 637 μm) supports the theory that large filter-feeders are more susceptible to mechanical interference [43,47]. Long filaments can clog the feeding apparatus, increasing handling time and forcing frequent rejection, which decreases energetic efficiency [48].

The superior grazing of *Macrothrix paulensis* on *Dolichospermum planctonicum* DPJ-07 suggests an ability to manipulate and fragment filaments. Similar mechanisms have been observed in *Macrothrix spinosa*, which could “cut” filaments to facilitate ingestion [25]. A study with tropical zooplankton also showed that small cladocerans can consume filamentous cyanobacteria, highlighting the existence of adaptive mechanisms [49]. Furthermore, the origin of the organisms is also a determining factor: both the cladocerans and the cyanobacteria studied were isolated from reservoirs with a history of *Dolichospermum* blooms. For instance, *Macrothrix spinosa* clones from eutrophic environments with a history of blooms were more tolerant to the toxic filamentous cyanobacterium *Raphidiopsis raciborskii* than clones from an oligotrophic environment [25]. This acquired tolerance, combined with an adapted feeding strategy, may have given *Macrothrix paulensis* a competitive advantage.

The intermediate feeding performance of *Ceriodaphnia silvestrii* suggests that this species may not possess the same handling adaptation as *Macrothrix paulensis*. A previous finding demonstrated that *Ceriodaphnia quadrangular* could feed selectively on cryptophytes, which are nutritionally superior, rejecting diatoms of similar size [50]. It is possible that *Ceriodaphnia silvestrii* is also better adapted to consuming high-quality single-cell particles, being moderately inhibited by filament morphology. Therefore, studying the grazing efficiency of different tropical cladocerans is important to better understand the interactions between zooplankton and cyanobacteria, especially regarding the potential of these organisms to control cyanobacterial blooms.

Comparisons between the two *Dolichospermum planctonicum* strains showed that even within the same cyanobacteria species, filament size is crucial for interaction with zooplankton grazers. The single-diet results are in line with studies showing cladocerans’ preference for shorter filaments. For instance, *Daphnia pulicaria* preferentially consumed *Planktothrix* filaments shorter than 100 µm [51]. It should be noted, however, that the *Dolichospermum planctonicum* strains tested in this study had larger average filament sizes than those in the previously cited study (235 and 637 µm).

*To what extent do the proportions of different* *D. planctonicum* *strains within a mixed diet, with a high-quality food source also present, influence the grazing dynamics of tropical cladocerans occupying diverse feeding niches?*

Mixed-diet experiments revealed a complex shift in feeding behavior characterized by an inversion in cladocerans’ feeding responses. While the shorter-filament strain *Dolichospermum planctonicum* DPJ-01 was consumed less than DPJ-07 in single diets, it was consumed significantly more in mixed diets with the chlorophyte *Monoraphidium capricornutum.* This reversal suggests that the mechanism limiting grazing on filamentous cyanobacteria might change drastically depending on the food context, that is, whether the cladoceran is in a situation where a nutritional alternative is available or not. The ability to handle different phytoplankton morphologies is therefore crucial to zooplankton and dependent on the dietary context [52].

Long *Dolichospermum planctonicum* DPJ-07 filaments represent a severe physical obstacle that not only hinders their own consumption but is likely to interfere with the capture of *Monoraphidium capricornutum* cells as well. To maximize the intake of high-quality food, cladocerans might actively avoid long filaments to maintain an unobstructed filtering apparatus [20,47]. Shorter filaments cause less mechanical interference and are thus less energetically costly to process, leading to incidental higher ingestion while filtering for green algae [47,48]. Although cyanobacterial proportion was not statistically significant in GLM analyses, the visual trend suggests a decline in total clearance as *Dolichospermum planctonicum* dominance increases, a pattern previously described with filamentous cyanobacteria and other tropical cladocerans like *Moina micrura* [4]. This trend may indicate that the mere presence of the filaments is already sufficient to cause inhibition. In addition, morphology may not be the only factor influencing the feeding behavior of zooplankton [20,42,43]. Secondary metabolites as well as nutritional inadequacy might have acted on the grazing responses observed [30,53]. Thus, the observed reduction in filtration rates may be a combined response to mechanical interference, low nutritional quality and the presence of repressor compounds in the DPJ-07 strain.

*What are the selective feeding patterns of tropical cladocerans with a history of cyanobacteria exposure when offered a choice between filamentous* *Dolichospermum planctonicum* *and the nutritionally superior green alga* *Monoraphidium capricornutum* *?*

Feeding selectivity analysis revealed a capacity for food discrimination in tropical cladocerans, which prioritize the consumption of the item with the highest nutritional value and appropriate size and actively avoid those of low quality. Strain, dietary proportion, and morphology are decisive factors for food selectivity. Cladocerans showed strong positive selection for *Monoraphidium capricornutum*, but also for cyanobacteria at lower proportions. The ability to discriminate against high-quality food in the presence of cyanobacteria is a crucial survival strategy during blooms [13,14]. However, we observed that this pattern tends to be reduced with the dominance of cyanobacteria. Although the persistence of small-sized cladocerans during cyanobacterial dominance has been reported in many waterbodies, our data highlight selectivity in small tropical cladocerans. These results represent an underexplored pattern that challenges traditional models of zooplankton–cyanobacteria interactions [13,14].

*Daphnia gessneri* exhibited the most extreme selectivity, strongly rejecting *Dolichospermum planctonicum* filaments. While this prioritizes high-quality items, it renders the species vulnerable during blooms, where mechanical interference results in low total food intake [23,47]. *Macrothrix paulensis* demonstrated a more flexible strategy. Its less pronounced avoidance of cyanobacteria, especially the short-filament strain, suggests an adaptation to exploit low-quality resources inaccessible to competitors [27]. This adaptation may be linked to specific filament handling mechanisms, such as the ability to fragment them [25]. *Ceriodaphnia silvestrii* once again demonstrated intermediate behavior among the cladocerans studied, with a tendency more similar to *Daphnia gessneri* than to *Macrothrix paulensis*, but with less capacity for food selection under the conditions studied.

The dietary proportion of cyanobacteria significantly influenced selectivity, with rejection being strongest at the 75% proportion. This is ecologically coherent: when high-quality food is abundant, the energetic cost of handling low-quality items makes their ingestion disadvantageous. Thus, organisms must become more selective to maximize net energy gain [13,54].

In summary, all the results observed in this study (single and mixed diets and selectivity) demonstrate a great diversity of grazing strategies of cladocerans on the same species of the bloom-forming filamentous cyanobacterium *Dolichospermum planctonicum*. The current understanding of cladocerans’ feeding behavior is mainly derived from studies of temperate and big cladocerans, such as *Daphnia* [13,22]. Our study brings information from small tropical cladocerans, which can be dominant during cyanobacterial blooms [23], and shows that these can vary significantly in grazing strategies, shifting preferences depending on food context in the environment.

In single-diet assays, grazing efficiency varied significantly across food types and concentrations. While the largest cladoceran such as *Daphnia gessneri* was the most efficient consumer of high-quality food, its clearance rates were severely inhibited by *Dolichospermum planctonicum*’s long filaments. Conversely, the smallest species, *Macrothrix paulensis*, exhibited higher tolerance to long filaments. The introduction of an alternative high-quality food source in the mixed diets triggered a shift in consumption patterns: the short-filament strain became the more ingested strain. This suggests a strategic shift toward maintaining mechanical efficiency in the presence of preferred prey.

All studied cladocerans exhibited selective feeding behavior, prioritizing nutritious algae and avoiding cyanobacteria. *Daphnia gessneri* displayed the strongest avoidance strategy, whereas *Macrothrix paulensis* proved to be more flexible, particularly with shorter filaments. These findings corroborate the hypothesis that different cladoceran species exhibit distinct grazing responses and that increasing *Dolichospermum planctonicum* biomass negatively affects clearance rates, with the most severe inhibition occurring in large-bodied species exposed to long filaments. Feeding selectivity in small-bodied tropical species may be a key adaptation for these animals in tropical environments with cyanobacteria dominance and/or blooms.

The results demonstrate that grazing on *Dolichospermum planctonicum* is a multifaceted interaction mediated by cladoceran body size, cyanobacterial morphology (filament size), and dietary composition. Our findings address a critical knowledge gap regarding trophic interactions in tropical freshwater ecosystems, which are frequently dominated by small-bodied cladocerans during cyanobacterial blooms. This study highlighted that in tropical ecosystems, zooplankton community structure can be shaped by phytoplankton quality and morphology, with direct implications for energy transfer and the potential for top-down control during bloom events.

## Figures and Tables

**Table 1:** Significant differences were detected by Tukey’s post hoc test in filtration rates in single-diet experiments considering the following factors: zooplankton, *Dolichospermum planctonicum* strains, and concentrations.

Zooplankton	Estimate	Standard Error	z	*p*
*Daphnia–Ceriodaphnia*	0.07	0.01	4.5	<0.0001
*Macrothrix–Daphnia*	−0.06	0.01	−4.2	<0.0001
**Strains**	**Estimate**	**Standard error**	**z**	***p***
DPJ-07–DPJ-01	0.04	0.01	2.8	0.01
MONO–DPJ-01	0.06	0.01	4.0	<0.001
**Concentrations**	**Estimate**	**Standard error**	**z**	***p***
0.5–0.125	−0.07	0.02	−3.552	0.0034
1–0.125	−0.06	0.02	−3.222	0.0110
2–0.125	−0.1	0.02	−6.136	<0.001
0.5–0.25	−0.09	0.02	−2.770	0.0443
2–0.25	−0.1	0.02	−5.354	<0.001
2–1	−0.06	0.02	−2.914	0.0295

**Table 2:** Significant differences were detected by Tukey’s post hoc test in clearance rates (CRBlue, CRGreen and CRTotal) in mixed-diet experiments considering the following factors: zooplankton, strains, and proportion of cyanobacteria in the diet.

CRBLUE				
Zooplankton	Estimate	Standard Error	z	*p*
*Daphnia–Ceriodaphnia*	0.4	0.1	2.64	0.02
**Strains**	**Estimate**	**Standard error**	**z**	***p***
DPJ-07–DPJ-01	−0.7	0.1	−5.0	0.0000004
**CRGREEN**				
**Zooplankton**	**Estimate**	**Standard error**	**z**	***p***
*Macrothrix–Ceriodaphnia*	−0.7	0.2	−3.4	0.001
*Macrothrix–Daphnia*	−1.1	0.2	−5.6	<0.001
**CRTOTAL**				
**Zooplankton**	**Estimate**	**Standard error**	**z**	***p***
*Daphnia–Ceriodaphnia*	0.5	0.1	4.3	0.0001
*Macrothrix–Daphnia*	−0.7	0.1	−6.1	0.0001
**Strains**	**Estimate**	**Standard error**	**z**	***p***
DPJ-07–DPJ-01	−0.5	0.1	−5.2	0.0000001

## Data Availability

The original contributions presented in this study are included in the article. Further inquiries can be directed to the corresponding authors.
